# PREVALENCE AND PREDICTORS OF DECISIONAL CONFLICT AMONG OLDER AFRICAN AMERICANS WITH ADVANCED KIDNEY DISEASE

**DOI:** 10.1093/geroni/igad104.2540

**Published:** 2023-12-21

**Authors:** Tyrone Hamler

**Affiliations:** University of Denver, Denver, Colorado, United States

## Abstract

Chronic kidney disease (CKD) is a major public health concern in the U.S., particularly among African Americans. African Americans are just under four more times likely to develop CKD than White individuals. Older African Americans are a vulnerable and understudied population in CKD literature, despite future projections of an increasingly diverse population of older adults. This study’s objectives were: (1) to determine the prevalence of decisional conflict for pre-dialysis older African Americans (2) whether perceived CKD knowledge, clinical characteristics (self-rated health, depression, anxiety, and number of comorbidities), and personal characteristics predicted decisional conflict. Study participants (N =125) were recruited from an outpatient nephrology clinic in a midwestern hospital and administered a telephone survey. Participants were African American, ≥ 50 years of age, pre-dialysis and diagnosed with Stage 4 or 5 CKD. A hierarchical ordinary least-squares (OLS) regression analysis was conducted to examine the relationship between variables. Participants reported mean scores of DC in the moderate range (M = 36.69). Approximately 42% (n =53) of patients’ decisional conflict scores were above 37.5, which is the cut-point for scores indicating uncertainty (O’Connor, 1995). Multivariate analyses indicated that DC was only significantly associated with perceived chronic kidney disease knowledge. Lower levels of perceived chronic kidney disease knowledge were associated with higher levels of DC. Findings indicated that DC is a critical area to continue to investigate among older African Americans with advanced CKD. Social workers are well-positioned to impact levels of knowledge in this population through working on interdisciplinary teams in medical settings.

